# Evaluating hospital performance in antibiotic stewardship to guide action at national and local levels in a lower-middle income setting

**DOI:** 10.1080/16549716.2020.1761657

**Published:** 2020-06-26

**Authors:** Jacob McKnight, Michuki Maina, Mathias Zosi, Grace Kimemia, Truphena Onyango, Constance Schultsz, Mike English, Olga Tosas-Auguet

**Affiliations:** a Nuffield Department of Medicine, University of Oxford, Oxford, UK; b Amsterdam University Medical Centres, University of Amsterdam, Amsterdam, The Netherlands; c KEMRI-Wellcome Trust Research Programme, Health Services Research Group, Nairobi, Kenya; d Amsterdam Institute for Global Health and Development, Amsterdam, The Netherlands

**Keywords:** Antimicrobial Resistance, Antibiotic Stewardship, rational drug use, AMR, LMIC, resistance, hospitals

## Abstract

**Background:**

Inappropriate use of antibiotics can lead to the development of resistant pathogens. Ensuring proper use of these important drugs in all healthcare facilities is essential. Unfortunately, however, very little is known about how antibiotics are used in LMIC clinical settings, nor to what degree antibiotic stewardship programmes are in place and effective.

**Objective:**

We aimed to record all Antibiotic Stewardship policies and structures in place in 16 Kenyan hospitals. We also wanted to examine the context of antibiotic-related practices in these hospitals.

**Methods:**

We generated a set of questions intended to assess the knowledge and application of antibiotic stewardship policies and practices in Kenya. Using a set of 17 indicators grouped into four categories, we surveyed 16 public hospitals across the country. Additionally, we conducted 31semi-structured interviews with frontline healthcare workers and hospital managers to explore the context of, and reasons for, the results.

**Results:**

Only one hospital had a resourced ABS policy in place. In all other hospitals, our survey teams commonly identified structures, resources and processes that in some way demonstrated partial or full control of antibiotic usage. This was verified by the qualitative interviews that identified common underlying issues. Most positively, we find evidence discipline-specific clinical guidelines have been well accepted and have conditioned and restricted antibiotic use.

**Conclusion:**

Only one hospital had an official ABS programme, but many facilities had existing structures and resources that could be used to improve antibiotic use. Thus, ABS Strategies should be built upon existing practices with national ABS policies taking maximum advantage of existing structures to manage the supply and prescription of antimicrobials. We conclude that ABS interventions that build on established responsibilities, methods and practices would be more efficient than interventions that presume a need to establish new ABS apparatus.

## Background

Antimicrobial Resistance (AMR) represents an existential threat to global health. The reduction of overuse and improper use of antibiotics through Antibiotic Stewardship (ABS) programmes is a cornerstone of WHO’s Global Action Plan (GAP) [1]. The GAP recognises that regulation is weak, that usage data is poor, and that the situation is made more difficult by a lack of evidence-based diagnosis and the proliferation of sub-standard antibiotics [1, p. 10]. Poor prescribing practices combine with low levels of infection prevention and control to encourage a faster spread of resistant pathogens in Low and Middle-Income Countries (LMICs) [2]. For exhample, 214,500 neonatal sepsis deaths worldwide are thought to be attributable to resistant pathogens, 52% of which are experienced in just five LMIC countries [3].

More positively, a recent review suggested that ABS interventions in LMICs can have a positive effect [4]. However, in many cases, studies of interventions were of low quality with a high degree of heterogeneity in results meaning that it is difficult to draw direct conclusions from the analysed literature. Two systematic reviews in higher-income settings also suggested that interventions can have a positive effect on ABS, but again, the heterogeneity of approaches makes it difficult to draw conclusions on what works best [5,6].

Few surveys have been conducted in LMIC contexts to show the level of implementation and effectiveness of existing ABS structures, thinking, and practices [7]. In addition to providing an important ‘baseline’ for future ABS interventions, implementation science highlights the importance of understanding context as a dynamic part of interventions [8]. If ABS is to become what May calls ‘an ongoing achievement’, we need to understand not only if an intervention can have an effect, but also why and what the contextual mechanisms are that enable it. Here we report work helping us to understand context and opportunities for intervention in Kenyan hospitals.

## Methods

### Study setting

In Kenya, the Ministries responsible for Health and Agriculture appointed a multi-sectoral Technical Working Group to develop national guidelines for the prevention and containment of antimicrobial resistance [9]. Kenya has a devolved system of government however, and provision of healthcare falls to the county governments who are responsible for any implementation of the AMR policies and plans.

The survey and interviews covered 16 public hospitals in Kenya and were conducted alongside related work investigating Infection Prevention and Control [10]. The hospitals are part of the Clinical Information Network (CIN) of the Kenya Ministry of Health. The CIN was set up to collate data from paediatric inpatient units to promote development and adoption of evidence-based clinical guidelines [11] and is coordinated by the Kenya Medical Research Institute (KEMRI) Wellcome Trust Research Programme. Six of the 16 facilities are located in areas of Kenya with a high malaria prevalence. The bed capacities across the 15 county facilities range between 120–550 beds with between 6 and 18 separate inpatient wards and between 5–26 consultants in total across all medical and surgical specialties [12]. The study hospitals are all public facilities. Although there are many private hospitals in Kenya (282/724, 39%) and missionary hospitals (93/724, 13%) public hospitals are thought to manage a majority of the demand (348/724, 48%) [13]. In one of the facilities, which acts as the national referral hospital, due to its size, we only assessed ABS arrangements in the paediatric and neonatal units. These wards were chosen because WASH should be a priority where there are particularly vulnerable patients and because the research team has long-standing professional relationships with the management of these wards.

### Survey preparation

We sought to identify an ABS survey tool that would sufficiently capture relevant aspects of ABS in the hospital context. We began with a desk review of available ABS policies in Kenya and internationally drawing particularly on the ‘UK’s NICE guidance as it offered practical guidance at the hospital level. NICE’s ‘Antimicrobial stewardship: systems and processes for effective antimicrobial medicine use’, was particularly useful in deriving a set of survey questions [14]. It promotes the use of prescription guidelines, a point further emphasised by NICE’s Infection prevention and control quality standards [15].

The NICE ABS guidance provides a ‘Baseline Assessment Tool’, but this is developed specifically for the UK NHS. We drew upon our experience in Kenya to adapt the tool to evaluate extant processes and structures, rather than only ABS/AMR-specific ones. Table 1 (supplementary materials) shows the references for each question asked. For each of the 17 questions, the surveyor scores a facility as either fully, partially or not meeting recommended targets as defined in the study’s standard operating procedures. An open text section also allows the data collector to record the logic behind the scores assigned. The ABS data collection tool (Supplementary Materials) was piloted in a hospital similar to the study hospitals and revised before actual data collection.

### Survey data collection

Data collection was conducted by 3 study teams each of 4–5 people in three different regions of the country: central Kenya (4 hospitals), Western Kenya (5 hospitals), and around the capital city (5 hospitals). The 3 study teams’ members received training from the investigators prior to the data collection process. For each hospital, upon receiving permission from the manager, the teams approached the head pharmacist. Across the 16 facilities, these were graduate pharmacists some of whom had specialised post graduate training in clinical pharmacy. The pharmacist was taken through the survey providing responses that were graded by the study data collection team into one of the three possible levels.

### Survey data analysis

Individual indicators were grouped within the 5 pre-specified ABS modules: leadership; accountability and expert support; supplies; monitoring and reporting; and policy and practice. For each of the 17 indicators ‘heat map’ colour codes were generated based on the indicator: does not meet (red); partially meets (orange); meets (green). In addition, we compute aggregate indicator scores by assigning numeric scores to each level. Does not meet the target, partially meets and meets target were assigned numeric scores 0, 1, and 2 respectively. Percentage scores were derived as a proportion of the sum of the numerators(individual indicator scores for each hospital) and dividing these by 32 which is the sum of denominators representing the maximum possible score if all hospitals met target for each of the indicators.

### Qualitative approach

Semi-structured interviews were conducted with hospital managers (e.g. medical directors, nursing and laboratory heads) and frontline health workers (e.g. consultants, medical and nursing officers) during the survey visits, in 7 of the 16 hospitals – sampled to ensure spread across different geographical locations represented by the study hospitals. The survey team included a team member from each hospital visited, and these individuals made the introductions and explained the research for each interview.

Interviews were conducted by the first, second, fourth and fifth authors in the hospital setting, using a semi-structured interview instrument that consisted of different, but strongly related questions to that of the survey [10,12]. All authors have experience with medical research in Kenya, but the first author is experienced in using qualitative methods and trained the others in the use of the semi-structured tool and general interviewing techniques following the ‘long’ or ‘ethnographic’ technique [16]. We conducted 31 interviews between November 2017 and March 2018 each lasting between 30 mins and 90 mins with no one refusing to be interviewed or dropping out and no repeats. We used both purposive and snowball sampling and were mindful of emerging important issues such as training and managerial support, and experience and aimed to reflect this diversity in our interviewee sampling strategy. Responsibilities for drug use are rarely defined by formal rules at the hospital level, but normative expectations around the roles of different staff are shared across hospitals in Kenya and well understood by the research team. As such, we targeted nurses and doctors but also spoke to pharmacists and laboratory technicians.

The semi-structured interview process provided opportunities to investigate emergent areas of interest, identified through discussions with the research team and review of transcripts, and this allowed us to move beyond areas where we had reached saturation and onto new areas. Interviews were conducted in a quiet place near the place of work with one or two interviewers and the interviewee. At the end of each interview, the respondent was asked to raise any issues that they felt were important but not addressed. We did not return transcripts to the respondents, but did check our findings with some hospital managers and the Ministry of Health and county stakeholders with whom we are connected.

The audio files were transcribed and uploaded into NVivo 12. Our methodological framing was exploratory and inductive, and though guided by initial discussions between the team and the stakeholders, adopted a grounded theory approach. Open codes were individually generated according to best practice in qualitative research by the 1st, 2nd and 4th authors [17]. Then we sought to match the findings of the survey with the themes that emerged from the qualitative analysis. The analysis presented below is organised so as to allow us to explore inductively derived issues within the broad categories used in the survey. This approach allows us to add context and detail to each of the survey questions.

All interview tools and information sheets are available on request. A Consolidated Criteria for Reporting Qualitative research (COREQ) checklist was completed and is included as an appendix, following a format provided by Tong et al. [18].

## Results

The survey was carried out at all 16 hospitals. The most senior pharmacist available during the visit was chosen to respond to the questions. For the qualitative interviewing, 31 respondents were interviewed using the semi-structured interview instrument that was adapted infection prevention guidelines by ward throughout the research to reflect our growing understanding of the key issues. Overall the indicator scores ranged between 28 and 69%. The indicator assessing availability of local speciality-specific antibiotic guidelines had the lowest overall performance. There was no significant difference in indicator performance between the hospitals in the high and low malaria-endemic zones.

**Figure 1.**
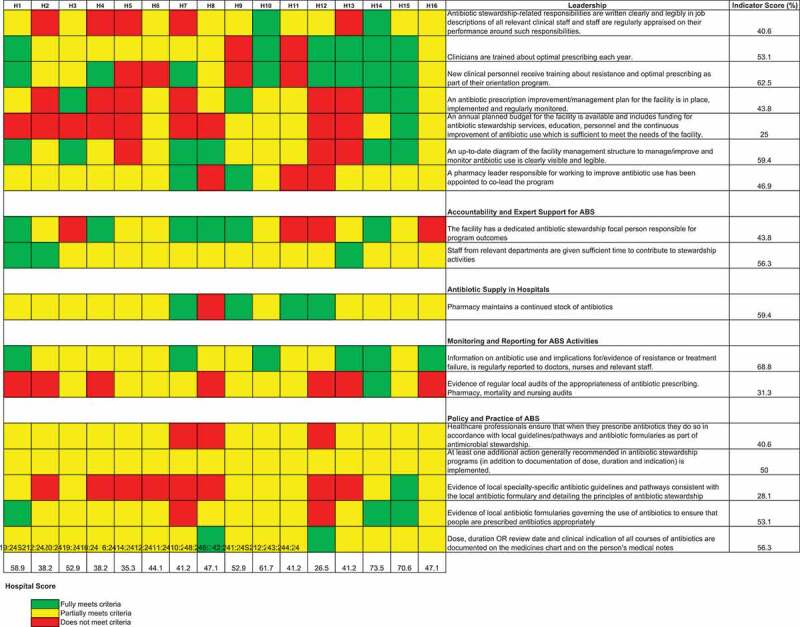
Performance based on 17 indicators grouped in 5 different domains is shown by the upper vertical bars. The right horizontal bars summarise the performance of each indicator across 16 hospitals. The tiles in the central grid are coloured according to the performance classification of each indicator in each hospital, as shown in the colour legend

### Leadership

Seven indicators assess leadership for ABS. As can be seen in Figure 1, there was mixed performance across the hospitals and the indicators, with H5 and H12 being low performers and H14 and H15 being highly compliant. Figure 1 also indicates that it is unusual for a hospital to have an annual planned budget for ABS.,

### Accountability and expert support for ABS

Figure 1 shows that while a person might be identified as being responsible for ABS, this was far from universal. Additionally, while staff were given some time to contribute to ABS activities, this was often not an official directive, but instead a general part of individual workers’ roles. This is explored more thoroughly in the qualitative section below.

Antibiotic Supply in Hospitals – this simple indicator shows that most hospitals, experienced difficulties in maintaining a full stock of antibiotics. Encouragingly, in 4 hospitals, antibiotic supply was not thought to be a problem.

### Monitoring and reporting for ABS activities

We aimed to understand if the pharmacists are able to track how specific antibiotics are being prescribed across hospital departments or disease conditions and also monitor the quality of prescriptions, e.g. posology, duration, and route of administration. Only 1 facility achieved the set targets for the two indicators assessed. The other facilities were deemed to partially meet the target because the pharmacists regularly attended the ward rounds within these departments and kept track of what and how antibiotics are being prescribed using this informal mechanism.

### Policy and practice of ABS

We assessed this through a set of five indicators. As can be seen in Figure 1, we note significant challenges with guideline availability and use as evidenced by a lack of specialty-specific guidelines

## Qualitative analysis

### ABS leadership

Most of the respondents we spoke to had not heard the phrase Antibiotic Stewardship. Thus, while they might understand the mechanism, they thought of ABS as a principle rather than a particular programme or area of work:

“So far no, I think mostly, the majority of the people are yet to be aware about ‘antimicrobial stewardship programme’. The majority, even if you do the survey currently, they are not even aware what this term means.”

Nurse and IPC focal person

This general lack of awareness meant that there were no specific budgets for ABS activities, and also, that there was no specific training for ABS. However, several hospitals demonstrated general leadership in the use of all drugs and who had managed to fund Continuing Medical Education (CME) and other forms of training focused on the use of antimicrobials. Pharmacists, while often not familiar with the phrase ‘Antibiotic Stewardship’, were familiar with the idea of ‘rational use’ of drugs in general and had sought to train other staff on this issue.

“Usually, we have CMEs in Hospital, and it’s [‘rational use of drugs’] an issue that is usually shared among hospital staffs.”

Pharmacist

While the interviews and survey identified a number of structures and resources that could be used for further work in ABS, it is important to note that leadership must attend to all problems, not just ABS. For example, where clinical pharmacists do exist, they are well-positioned to lead training on this issue have other issues to deal with such as intern clinical officers unsure of general drug use:

“correcting outpatients prescriptions, that actually is the largest challenge … because for the staff that’s there they have a very high staff turnover, and patient and especially they have a new clinical officers who are on training and they rotate therefore I think a span of the three months, and they are fresh, so there is a lot of hiccups on dosing, right indications. That is our biggest challenge.”

Pharmacist

### Accountability and expert support for ABS

It is important to recognise potentially untapped resources and existing organisational structures in place that could improve prescription practices. The presence of clinical pharmacists was key because they understood the general principle and could address inappropriate use of antibiotics where they accepted this responsibility. Thus, in the more effective hospitals, we found evidence of active and capable medicines and therapeutics committees.

“There’s also a committee in the hospital actually they have formed that that group. And they normally actually with the … they involve a lot of departments. So, in the lab we have one person who normally attend those meetings where they’re taught actually on how the drugs are supposed to be issued.”

Senior Nurse

But sometimes, even when a medicines and therapeutics committee is in place and has good representation from different areas of the hospital, underfunding and organisational difficulties may mean that tight control of ABS is beyond them.

“We don’t have a very healthy committee, like a stewardship to follow up to see this patient has been on the antibiotics for how long? Because sometimes you can have a prescription of a patient who has been on ceftriaxone for more than ten days – to me, I don’t think it is proper”

Pharmacist

It is also important to recognise the broader spectrum of prescribers working in Kenyan hospitals. Some nurses, clinical pharmacists and most commonly, clinical officers who influence prescribing or directly prescribe in Kenya appear to be excluded from decision making and training.

“I think there are there are some meeting for therapeutic committee and maybe that is where they discuss these things. And then, there’s another committee they call doctors meetings. You know not everybody is a doctor in this hospital, there’s clinical officers and they are prescribers, they have pharmaceutical technologists who dispense medicine and they’re not doctors, so the, we feel that there’re a group that are left without understanding what is going on in terms of therapeutic committee reports – they don’t get those reports.”

Lab Manager

A further governance issue detracting from standardised usage of antibiotics is failure to restrict access of pharmaceutical sales people who were obviously successful at promoting the use of their drugs within certain hospitals:

“Maybe with the influence of the med rep and then the many antibiotics they were prescribing. So as a committee, we thought maybe you could narrow these antibiotics”

Pharmacist

### Qualitative analysis of antibiotic supplies

The interviews confirmed that the supply of antibiotics was sometimes broken and though there were clear differences across the sites surveyed, most respondents recognised this as a problem.

“Stockouts are very often. Specific antibiotics, like even now I will tell you if a patient needs meropenem currently, we don’t even have a single vile yeah … Sometimes it causes delay of treatment because now if a patient needs maybe meropenem, they will assume maybe they’ve given this patient maybe ceftriaxone for a while and now the consultants or the clinician are thinking maybe this patient needs to be changed go get meropenem from the pharmacy. At that point they will not get, because at that point is when we initiate now to the process of procuring meropenem. So that might delay treatment for one, or two days, which is not good, yeah because of the protocol also in procurement issues, yeah.”

Pharmacist

In one case, a respondent hinted that supply problems were related to deals being done with improper or ineffective suppliers.

“They decided to award most of the tenders to the local suppliers. So that had been the challenge because actually the local suppliers, most of them don’t have the capacity to supply the items and sometimes the quality of the drugs they are supplying, we are not able to assure the quality. So sometimes it’s politics ehh? So yes, it’s beyond us.”

Pharmacist

Such problems can lead to breaks and delays in treatment which may contribute to the development of AMR.

“If you really need that drug, for example, if it is something that I want to use like I have decided this patient have meningitis and I want to use Ceftriaxone, now Ceftriaxone runs out by day 2, so the patient buys, in a hospital the patient buys … Yes, are you seeing the problems that we have. So, they must have someone they are sending to go and bring. That somebody might take two days to bring that drug or they tell you they don’t have money because that is the other thing – they just tell they don’t have money. And you can look at them and you can see clearly, they don’t have money.”

Paediatrician

A further important finding suggests that clinician prescribing behaviour changes when faced with supply shortages. This has particular implications for people who are adjudged to be unable to afford certain drugs

“Yes, that one you will do, that one you will definitely have to do. I mean like now if you tell someone like I want to treat someone, let me say someone has upper respiratory tract infection and I want to give the Amoxil and I really want to, I really … me I believe in the original, where I want them to get the original Amoxil from GSK and the person has Ksh 200. What are you going to do? You just tell them to go and buy whatever, I mean with the money you have go and buy what are they called? You know those bad generic and you let them do that because what are you going to do? They don’t have money, you cannot buy for them, and you are not giving them a solution. So, you give them a solution according to what they have.”

Paediatrician

Hence, poorer patients seem to be more likely to be prescribed medicines that are known to be less effective, to be unable to afford full treatment courses and also to find drugs of limited or nil pharmaceutical utility.

### ABS monitoring and reporting

As stated above, most structures and professionals that deal with ABS do so as part of other work, and not due to a particular ABS programme in place in the hospital. In many locations, there was a practice of the pharmacist advising clinicians on drug use. This may involve occasional visits to the ward to provide advice or seeking out particular doctors to discuss cases.

“Sometimes we send the patient back, or sometimes we call the clinician.

Clinical pharmacist

“But once in a while the pharmacist tends to come in the hall, you know, they come to the ward sometimes and they will just … ‘why are you giving this one, why are you not giving this?’

… Even simple things like dosages, they will always call you back from the ward, you come to the pharmacy, you are told what is supposed to be the right dosage, yeah.”

Medical officer

In sum, however, the auditing practices were very weak, there was little use of actual data and most processes in place were ad hoc, and involved pharmacists reacting to particular cases rather than being systematic about usage.

### Qualitative analysis of ABS policy and practice

We identified positive policies and practices in place that were not specifically for ABS, but generally supported ABS. Firstly, the staff we spoke to understood the usefulness of formularies and there seemed to be a history of attempts to implement them. Secondly, the large-scale adoption and application of the national paediatric guidelines show that it is possible to change and standardise the use of antibiotics in this context.

Our respondents identified formularies as addressing the issues we raised concerning ABS:

“So, we, we have a ceiling from where you can choose which antibiotics to prescribe, so we have a formulary.”

Medical Superintendent

However, in some locations, our respondents were not sure if they were in active use and suggested that their introduction had stalled:

“So yes, if it is formulary there is one that it is being developed. Did they finish developing it? I am not sure. But there is one that I had been called to actually develop. I am not sure how far it went.”

Paediatrician

“I remember … actually from … a few years back because I’ve been here for a while like almost five years, we started developing a hospital formulary for a whole week, customised hospital formulary.”

Pharmacist

Additionally, we found evidence that where a formulary was not applied, there was space for differential prescriptions, based not on the specific condition, but rather on the patient’s insurance status:

“P: If you go to our pharmacies, you will realise that we have separated patients, some NHIF clients and others. Essentially, we should just have a uniform formulary that is working for all of them.

I: You mean that if I got you right that if patient is under insurance and others paying cash, you separate the formulary they receive … ?

P: They are likely to get different products sometimes.”

Medical superintendent

The situation was similar with guidelines. Our respondents recognised the importance of guidelines. They pointed to the success of the paediatric guidelines, but most often suggested that other guidelines were not in use anywhere else in the hospitals we visited.

“Yah that one would vary, I think it varies for example we have a paediatric protocol that have guidelines on how to use antibiotics. Like this one should not be used in combination with this one, use these number of days. Maybe that, maybe that is what we have but in other wards no there is no guideline. Maybe it is present in pharmacy, maybe pharmacy have a copy of some sort but I am not aware.

Medical officer

No, it is not there but in paediatric wards those ones I know they are there, but in the wards, I know there is none in the surgery ward. I don’t know if they have the guideline, but they use their phones to look at what to give for how many days.”

Nurse

In the absence of a formulary or guideline, clinicians had a variety of coping mechanisms. Commonly, they searched the internet for answers, but it was not clear precisely what resources they were drawing on when they did this:

“They use the internet and google”

Nurse

Similarly, respondents also pointed to the importance of their training.

“Yes, there is guidelines in this hospital, there … I haven’t seen a guideline but back at the medical school and our training here, you need to have a justification for giving antibiotics, you don’t give everybody antibiotic”

Medical officer

And of consultants to whom they are currently or previously ‘apprenticed’:

“No [we do not follow guidelines] but we have our consultant. Our consultants who are really aggressive on when and what antibiotics to be given. When to change from first-line antibiotics when … how do you decide to go on to second line and when to go to on third line or when to refer or when to do culture? Yes, we have our consultants who are very keen, yes.”

Clinical officer

“Okay, for me like I can said, I still use the protocols for my former, for my former institution”

Medical officer

## Discussion

Recent work has highlighted the need to understand the state of antibiotic stewardship in LMICs [2,3,19] and has sought to provide standardised ways of approaching this important topic [7]. While evidence interventions can be effective in LMIC contexts exists, we lack clear benchmarks on current practice more broadly. Unfortunately, but perhaps not surprisingly, for all but one of the hospitals we visited in Kenya, there was no official recognition of ABS nor a specific programme in place to deal with this issue. Moreover, even senior pharmacists were not familiar with the phrase, though they were familiar with the more general idea of ‘rational use’ of drugs. Hence, given that the ABS elements of the 2017–2022 Kenyan national AMR policy [9] are yet to be implemented by county governments in regional hospitals, all survey questions that sought specific ABS structures and practices were likely to be scored negatively.

Despite this, our approach allowed us to understand the current ABS ‘lay of the land’ and identify extant structures and resources that are being used, or could be used, to control the use of antibiotics. Our findings serve to remind those interested in ABS, that antibiotics are well-established, crucial tools in all health systems, and so even without a specific ABS programme, their supply and use is already a matter of concern to a variety of health systems structures and professionals. In essence, antibiotics have social lives [20] and in many ways, can be seen as infrastructure [21]. Understanding and accepting the existing culture of antibiotic use for different geographies is key to interventions in this area [22].

Many of these issues we uncovered are linked not only to the specific supply and use of antimicrobials, but also to a wide range of health system failings. A lack of effective human resources; a lack of auditable data; problems in changing clinician behaviours; and shortages of supplies and budgets are all chronic issues that undermine many aspects of healthcare with ABS being but one of them. The origins and causes of these core issues are complex and beyond the remit of any single ABS intervention but important to tackle if ABS is to be effective.

Our work illuminates how the low ABS performance captured in our survey may contribute to AMR. Firstly, inadequate supplies can lead to rationing of medicines and stark choices regarding the patient’s ability to pay [23]. Clinicians make value judgements based on class and their perception of a patient’s ability to pay for drugs outside of the hospital. Secondly, where patients are adjudged to be poor by a clinician, they may prescribe a cheaper, though possibly less effective drug or a shorter course (A known behaviour for pharmacists – [24]). Thirdly, in sending patients out of the hospital to find medicines in the private sector, the clinician understands that this may lead to delays in treatment and doubts as to whether the drugs the patient or their carer procures are in fact real and effective rather than fakes or cheap copies. Fourthly, we note a converse effect where a clinician may be inclined to prescribe more expensive medicines where a patient makes it known that they have national or private insurance.

These issues are sometimes compounded by other long-standing health system failings. For example, the regular supply of medicines of dubious quality leads to a lack of trust in antibiotics, while the minimal availability of effective microbiological diagnostics, even in large hospitals [25,26], means that doctors cannot be sure what pathogen they are treating. This could result in a situation where a clinician may decide that the reason a course of antibiotics has failed to have the desired effect is due to drug quality rather than AMR.

This strong link to the private sector and doubts of the quality of medicines reminds us that hospitals are not biomedical vacuums where we can assume the actions of clinicians to be uniform across different geographies, but rather organisations entwined in local social and cultural milieus [27] and that the use of antibiotics in this setting is always connected to, rather than isolated from, the complex, broad social system of antibiotic use and ‘misuse’ [28].

The survey and related interviews also illuminate opportunities for improved ABS in Kenya’s hospitals. It was apparent that in paediatrics, the widely disseminated national guidelines [29] were the dominant influence suggesting discipline-specific guidelines can influence antibiotic use. Additionally, though formulary processes were moribund in the hospitals we visited, this was a well-recognised and potentially important way of guiding practice that could be reinstated.

We also noted for the hospitals in which they were active, medicines and therapeutics committees were powerful and influenced not only local prescribing patterns but were also involved in procurement and ensuring the regular supply of drugs to the facility. This is a particularly important point – in LMICs, more people die due to a lack of medication than to resistant pathogens [3]. While recent work has sought to set targets for reducing the use of antimicrobials, most LMICs are well below the new targets [2] suggesting we should help the patients who need antibiotics to access them in addition to reducing usage in those who do not.

Further, the study suggested that while few in number, clinical pharmacists are respected for their ability to provide suitable advice to frontline prescribers on the appropriate use of antibiotics and could play an important role in ABS programmes [30].

## Limitations

Our survey tool was derived from available UK documents but was not formally validated and is not as comprehensive as the checklist recently derived by Pulcini et al. [7] although the 4 section, 17 item checklist we used significantly overlaps with this 7 section 29 item list. Secondly, the selection of the hospitals was defined by participation in a clinical information network and they may not be representative of all Kenyan hospitals. However, we feel the combination of a survey tool and multiple interviews have enabled us to provide important insights into the current status of ABS in Kenyan hospitals that could inform more specific efforts to promote ABS practices that are suited to this context.

## Conclusion

Our findings perform three important functions: the survey offers a first attempt at benchmarking existing ABS practice in Kenyan public hospitals; the qualitative interviews provide detailed insights into the issues that affect antibiotic prescribing; and our analysis finds that many existing structures could be reinvigorated to improve ABS in Kenya. While there has rightly been a lot of attention given to the importance of ABS in reducing AMR, we should recognise that the use and misuse of antibiotics is determined by long-standing features of health systems. Recognising these as a prelude to initiating ABS programmes may be important to their success in limiting AMR in LMIC contexts.

## Supplementary Material

Supplemental Material

Supplemental Material

## Paper context

While an urgent case has been made for instituting ABS policies globally, little is known about which, if any, of these policies have been implemented in lower and middle-income economies. Our research explores the state of ABS in Kenya and suggests that while there were almost no ABS-specific policies in place, there were many long-standing structures and practices which governed the use of antibiotics. ABS interventions should be based on existing practices in each context.
