# Supplementary material for: Evaluating hospital performance in antibiotic stewardship to guide action at national and local levels in a lower-middle income setting
Source: Glob Health Action. 2020 Jun 26;12(Suppl):1761657. doi: 10.1080/16549716.2020.1761657 (PMC7782734; doi:10.1080/16549716.2020.1761657)
Supplement: Supplemental Material [file ZGHA_A_1761657_SM8074.docx]

| **#** | **Indicator** | **Reference** | **Sample text from Reference** |
| --- | --- | --- | --- |
| 1-4 | WASH FAST Questions above |  |  |
|  | LEADERSHIP |  |  |
| 5.1 | An up-to-date diagram of the facility management structure to manage/improve and monitor antibiotic use is clearly visible and legible | Antimicrobial stewardship: systems and processes for effective antimicrobial medicine use.  [1] | “Establish an antimicrobial stewardship programme, taking account of the resources needed to support antimicrobial stewardship across all care settings.” |
| 5.2 | An annual planned budget for the facility is available and includes funding for antibiotic stewardship services, education, personnel and the continuous improvement of antibiotic use which is sufficient to meet the needs of the facility. | Not considered by NICE ABS guidelines but included due to local relevance |  |
| 5.3 | An antibiotic prescription improvement/management plan for the facility is in place, implemented and regularly monitored. | NICE, Infection prevention and control  [2] | "Commissioners ensure that they commission services that have antimicrobial stewardship initiatives and in which people are prescribed antibiotics in accordance with local antibiotic formularies." |
| 5.4 | New clinical personnel receive training about resistance and optimal prescribing as part of their orientation program | NICE, Antimicrobial stewardship quality standard 121  [3] | “All prescribers of antimicrobials should have sufficient and appropriate training and competencies to deliver the actions and interventions described in the quality standard”. |
| 5.5 | Clinicians are trained about optimal prescribing each year. | Antimicrobial stewardship: systems and processes for effective antimicrobial medicine use  [1] | “providing education and training to health and social care practitioners about antimicrobial stewardship and antimicrobial resistance” |
| 5.6 | Antibiotic stewardship-related responsibilities are written clearly and legibly in job descriptions of all relevant clinical staff and staff are regularly appraised on their performance around such responsibilities. | Antimicrobial prescribing and stewardship competencies, ARHAI, Public Health England (approved by NICE)  [4] | "The principles and practice of the prevention and control of infection, and the need to have this reflected in individual job descriptions." |
| 5.7 | Staff from relevant departments are given sufficient time to contribute to stewardship activities | Not considered by NICE ABS guidelines but included due to local relevance |  |
|  | ACCOUNTABILITY AND EXPERT SUPPORT |  |  |
| 5.8 | The facility has a dedicated antibiotic stewardship focal person responsible for program outcomes | NICE recommends a multidisciplinary team, but a single focus person more attainable for this context. |  |
| 5.9 | A pharmacy leader responsible for working to improve antibiotic use has been appointed to co-lead the program | 1.1.8, Antimicrobial stewardship: systems and processes for effective antimicrobial medicine use, NICE Guideline 2015  [1] | "Organisations establishing antimicrobial stewardship teams should ensure that the team has core members (including an antimicrobial pharmacist and a medical microbiologist) and can co-opt additional members depending on the care setting and the antimicrobial issue being considered. " |
|  | SUPPLIES |  |  |
| 5.1 | Pharmacy maintains a continued stock of antibiotics | Not considered by NICE ABS guidelines but included due to local relevance |  |
|  | MONITORING AND REPORTING |  |  |
| 5.11 | Evidence of regular local audits of the appropriateness of antibiotic prescribing.  Pharmacy, mortality and nursing audits | 1.1.3 Antimicrobial stewardship: systems and processes for effective antimicrobial medicine use  [1] | "Consider including the following in an antimicrobial stewardship programme: integrating audit into existing quality improvement programmes." |
| 5.12 | Information on antibiotic use and implications for/evidence of resistance or treatment failure, is regularly reported to doctors, nurses and relevant staff. | Not considered by NICE ABS guidelines but included due to local relevance |  |
|  | POLICY AND PRACTICE |  |  |
| 5.13 | Dose, duration OR review date and clinical indication of all courses of antibiotics are documented on the medicines chart and on the person’s medical notes | Not covered by NICE ABS guidance, but perhaps covered by their other policies. |  |
| 5.14 | Evidence of local antibiotic formularies governing the use of antibiotics to ensure that people are prescribed antibiotics appropriately | NICE, Infection prevention and control, [2] | "Commissioners ensure that they commission services that have antimicrobial stewardship initiatives and in which people are prescribed antibiotics in accordance with local antibiotic formularies." |
| 5.15 | Evidence of local specialty-specific antibiotic guidelines and pathways consistent with the local antibiotic formulary and detailing the principles of antibiotic stewardship | Not covered by NICE ABS guidance, but perhaps covered by their other policies. |  |
| 5.16 | At least one additional action generally recommended in antibiotic stewardship programs (in addition to documentation of dose, duration and indication) is implemented | NICE, Infection prevention and control, [2] | Full list used for ‘additional actions’ in questionnaire provided within quality statement 1. |
| 5.17 | Healthcare professionals ensure that when they prescribe antibiotics they do so in accordance with local guidelines/pathways and antibiotic formularies as part of antimicrobial stewardship. | Antimicrobial stewardship: systems and processes for effective antimicrobial medicine use  [1] | “1.1.24 When prescribing antimicrobials, prescribers should follow local (where available) or national guidelines on: prescribing the shortest effective course the most appropriate dose and route of administration” |

*Table 1. The table shows how our ABS survey questions are linked to relevant NICE guidelines and policy documents or where they were derived so as to address relevant contextual issues*

## References

1. NICE. Antimicrobial stewardship: systems and processes for effective antimicrobial medicine use. National Institute for Health and Care Excellence; 2015.

2. NICE. Infection prevention and control. National Institute for Health and Care Excellence; 2014.

3. NICE. Antimicrobial stewardship. National Institute for Health and Care Excellence; 2016.

4. ARHAI. Antimicrobial prescribing and stewardship competencies. In: Infections DoHECoARaHCA, editor.: Public Health England; 2013.
