# Supplementary material for: Evaluating hospital performance in antibiotic stewardship to guide action at national and local levels in a lower-middle income setting
Source: Glob Health Action. 2020 Jun 26;12(Suppl):1761657. doi: 10.1080/16549716.2020.1761657 (PMC7782734; doi:10.1080/16549716.2020.1761657)
Supplement: Supplemental Material [file ZGHA_A_1761657_SM8073.docx]

**Standard Operating Procedures for Antibiotic Stewardship Survey**

Below is a brief description of what the antibiotic stewardship survey entails. You can use the information provided here to explain to health workers what the survey is about as you obtain consent.

**Antibiotic Consumption Survey**

The main aim of this survey is to understand some of the dynamics of antibiotics use in the hospital in terms of the leadership, accountability and expert support for antibiotic use, supply of antibiotics, monitoring and reporting on antibiotic use and finally looking at the supply of antibiotics in the hospitals.

Using these main themes, I intend to discuss with you and to learn more about how antibiotics are used in this hospitals.

Please remember it is important to advise the health workers to provide honest answers about what HAPPENS in real life and not answers that they think are ‘correct’.

**The health workers to discuss with are a Medical officer, Chief pharmacist and the chief nurse**

**Below is the SOP**

1. Introduce yourself to the health worker and explain that the hospital has been included in a survey of hospitals being led by MoH and conducted with KWTRP.
2. Explain that part of this survey is to seek the views of the health workers on antibiotic use in the hospital. This is complementary to the data being collected by the antibiotic consumption survey
3. Once consented please go ahead with the interview.

| **ANTIBIOTIC STEWARDSHIP** | | **Comments** | **Meets target**  **(+++)** | **Partially meets target**  **(++)** | | **Does not meet target**  **(+)** | | **Notes** |
| --- | --- | --- | --- | --- | --- | --- | --- | --- |
| **Leadership** | | | | | | | | |
| **5.1**  **(F)** | **An up-to-date diagram of the facility management structure to manage/improve and monitor antibiotic use is clearly visible and legible.**  Questions  Do you have a facility management team that helps manage/improve and monitor antibiotics?  Who are the members of the team?  How often do they meet?  Do they have minutes  Is it in writing diagram?  Is it implemented.? | Structure – includes any committees |  |  | |  | |  |
| **5.2**  **(F)** | **An annual planned budget for the facility is available and includes funding for antibiotic stewardship services, education, personnel and the continuous improvement of antibiotic use which is sufficient to meet the needs of the facility.**  Questions  Do you have an annual planned budget for the facility that includes funding for antibiotic stewardship services?  Education?  Personnel?  Continuous improvement of antibiotic use? | Education includes CME, morality meetings or formal trainings |  |  | |  | |  |
| **5.3**  **(F)** | **An antibiotic prescription improvement/management plan for the facility is in place, implemented and regularly monitored.**  Questions  Is there any antibiotic prescription improvement/management plan for the facility?  Evidence of documentation?  Is it implemented and regularly monitored? | Is there any plan to improve how antibiotics are prescribed |  |  | |  | |  |
| **5.4**  **(W)** | **New clinical personnel receive training about resistance and optimal prescribing as part of their orientation program.**  Questions  Do new clinical personnel receive guidance or training about resistance and optimal prescribing from the hospital?  Is it as part of their orientation program? |  |  |  | |  | |  |
| **5.5**  **(W)** | **Clinicians are trained about optimal prescribing each year.**  Questions  Are clinicians trained about optimal prescribing?  Optimal- Correct documentation on the treatment sheets, updating prescriptions?  How often? How many times in a quarter? | Training would include CME, formals courses or the mortality meetings |  |  | |  | |  |
| **5.6**  **(F)** | **Antibiotic stewardship-related responsibilities are written clearly and legibly in job descriptions of all relevant clinical staff and staff are regularly appraised on their performance around such responsibilities.**  Questions  Do you have a job description?  Are antibiotic stewardship-related responsibilities (including IPC) are written clearly and legibly in job descriptions of all relevant clinical staff?  Are staff regularly appraised on their performance around such responsibilities? | Responsibility included infection prevention and control |  |  | |  | |  |
| **5.7**  **(W)** | **Staff from relevant departments are given sufficient time to contribute to stewardship activities**  Questions  Are staff from relevant departments included in the IPC committee?  Are they given sufficient time to contribute to stewardship activities? |  |  |  | |  | |  |
| **PART B: ACCOUNTABILITY & EXPERT SUPPORT** | | | | | | | | |
| **5.8**  **(F)** | **The facility has a dedicated antibiotic stewardship focal person responsible for program outcomes**  Questions  Does the facility have an antibiotic stewardship focal person?  Does the person have sufficient time and resources to carry out the duties? |  |  |  | |  | |  |
| **5.9**  **(F)** | **A pharmacy leader responsible for working to improve antibiotic use has been appointed to co-lead the program**  Questions  Is there a pharmacy leader responsible for working to improve antibiotic use has been appointed to co-lead the program?  Is there a pharmacy team member dealing with ABS?  Does the person have sufficient time and resources to carry out the duties? | Any pharmacy member looking at antibiotic prescriptions in terms of accuracy and safety? |  |  | |  | |  |
| **PART C: SUPPLIES** | | | | | | | | |
| **5.10**  **(F)** | **Pharmacy maintains a continued stock of antibiotics**  Questions  Does the pharmacy maintain a continued stock of antibiotics?  Do the common antibiotics run out of stock?  How many times a year?  In the past three months?  For how long do these shortages last?  What to do if there is no supply? | Use the antibiotics on the essential drug lists |  |  | |  | |  |
| **PART D: MONITORING AND REPORTING** | | | | | | | | |
| **5.11 ***  **(F)** | **Evidence of regular local audits of the appropriateness of antibiotic prescribing.**  **Pharmacy, mortality and nursing audits**  Questions  Are there regular local audits of the appropriateness of antibiotic prescribing?  How often are these audits carried out?  Any documentation from these audits | Audits include the mortality meetings or any other meeting convened to discuss antibiotics  Frequency in the last quarter |  |  | |  | |  |
| **5.12**  **(F)** | **Information on antibiotic use and implications for/evidence of resistance or treatment failure, is regularly reported to doctors, nurses and relevant staff.**  Questions  Does the pharmacist communicate with the clinicians?  How?  About what?  For the clinicians – are there times you have reservations about the quality of the antibiotics you prescribe?  What do you do in these cases?  Is information on antibiotic use and implications for/evidence of resistance or treatment failure regularly reported to doctors, nurses and relevant staff?  How often does this reporting happen? |  |  |  | |  | |  |
| **PART E: POLICY AND PRACTICE** | | | | | | | | |
| **5.13**  **(W)** | **Dose, duration OR review date and clinical indication of all courses of antibiotics are documented on the medicines chart and on the person’s medical notes**  Questions  Are Doses, duration OR review dates and clinical indications of all courses of antibiotics documented on the medicines chart and on the person’s medical notes? | observation of some treatment charts in the wards |  |  | |  | |  |
| **5.14 ***  **(W)** | **Evidence of local antibiotic formularies governing the use of antibiotics to ensure that people are prescribed antibiotics appropriately**  Questions  Do you have local antibiotic formularies governing the use of antibiotics to ensure that people are prescribed antibiotics appropriately?  Any document available?  How often are these reviewed? | Confirm with the PPS data |  |  | |  | |  |
| **5.15**  **(F)** | Evidence of local specialty-specific antibiotic guidelines and pathways consistent with the local antibiotic formulary and detailing the principles of antibiotic stewardship  Confirm if these are regularly updated | Look for physical copies from any of the respondents  Any update in the last 2 years? |  |  | |  | |  |
| **5.16 *** | At least one additional action generally recommended in antibiotic stewardship programs (in addition to documentation of dose, duration and indication) is implemented.   1. Prescribe single-dose antibiotics for surgical prophylaxis if antibiotics have been shown to be effective. 2. Consider a no, or delayed, antibiotic strategy for acute self-limiting upper respiratory tract infections. 3. Obtain cultures, changing therapy to effectively treat resistant pathogens, and stopping antibiotics when cultures suggest an infection is unlikely. 4. Review the clinical diagnosis and the continuing need for antibiotics by 48 hours from the first antibiotic dose and make a clear plan of action. 5. Consider the options of the action plan after review: Stop, Switch Intravenous to Oral, Change, Continue, and Outpatient Parenteral Antibiotic Therapy. 6. Clearly document the review and subsequent decision in the person's medical notes. 7. **Limit prescribing over the phone to exceptional cases.** 8. Use simple generic antibiotics if possible. Avoid broad-spectrum antibiotics if narrow-spectrum antibiotics remain effective. 9. Avoid widespread use of topical antibiotics. | **Sample treatments sheets? /PPS data** |  |  | |  | |  |
| **5.17 ***  **(W)** | Healthcare professionals ensure that when they prescribe antibiotics they do so in accordance with local guidelines/pathways and antibiotic formularies as part of antimicrobial stewardship. | **From the PPS data** |  |  | |  | |  |
| Number of indicators meeting targets for **ANTIBIOTIC STEWARDSHIP** | | | | | **+++** | |  | |
| Number of indicators partially meeting targets for **ANTIBIOTIC STEWARDSHIP** | | | | | **++** | |  | |
| Number of indicators not meeting targets for **ANTIBIOTIC STEWARDSHIP** | | | | | **+** | |  | |
| \| **NOTES** \| \| --- \| \| **5.11**  Audits include determining if prescribers have: accurately applied diagnostic criteria for infections; prescribed recommended agents for a particular indication; documented the indication and planned duration of antibiotic therapy amongst other. \| \| **5.14**  A local antibiotic formulary is a local policy document produced by a multi-professional team, usually in a hospital trust or commissioning group, combining best evidence and clinical judgement. A local antibiotic formulary is defined as 'the output of processes to support the managed introduction, utilisation or withdrawal of healthcare treatments within a healthy economy, service or organisation. Local policies often limit the antibiotics that may be used to achieve reasonable economy consistent with adequate cover, and to reduce the development of resistant organisms. A policy may indicate a range of antibiotics for general use (i.e. for managing common infections), and permit other antibiotics only on the advice of the focal person/s responsible for antibiotic stewardship. \| \| **5.16**  **In addition to**   1. Documenting clinical indication, duration or review date, route and dose on the medicines chart and in the person's medical notes. 2. Not starting antibiotics without clinical evidence of bacterial infection. 3. Prescribing an antibiotic only if there is likely to be a clear clinical benefit (i.e. only when needed) 4. Using local guidelines to start prompt, effective antibiotic treatment if there is evidence or suspicion of bacterial infection,   **The approach to prescribing in line with the principles of antimicrobial stewardship also include to:**   1. Prescribe single-dose antibiotics for surgical prophylaxis if antibiotics have been shown to be effective. 2. Consider a no, or delayed, antibiotic strategy for acute self-limiting upper respiratory tract infections. 3. Obtain cultures, changing therapy to effectively treat resistant pathogens, and stopping antibiotics when cultures suggest an infection is unlikely. 4. Review the clinical diagnosis and the continuing need for antibiotics by 48 hours from the first antibiotic dose and make a clear plan of action. 5. Consider the options of the action plan after review: Stop, Switch Intravenous to Oral, Change, Continue, and Outpatient Parenteral Antibiotic Therapy. 6. Clearly document the review and subsequent decision in the person's medical notes. 7. Limit prescribing over the phone to exceptional cases. 8. Use simple generic antibiotics if possible. Avoid broad-spectrum antibiotics if narrow-spectrum antibiotics remain effective. 9. Avoid widespread use of topical antibiotics.   **Assess whether at least one of the above actions (see 1-9) is implemented.** \| \| **5.18**  **When assessing adherence to prescription guidelines/pathways consistent with antibiotic formularies, consider adherence to the following principles:**   1. Not starting antibiotics without clinical evidence of bacterial infection. 2. Prescribing an antibiotic only if there is likely to be a clear clinical benefit (i.e. only when needed)   **This indicator should be assessed through interviews, observation of practice, and review of the data resultant from the point prevalence survey of antibiotic consumption against local formularies, guidelines and/or pathways.** \| | | | | | | | | |
